# Sports Nutrition Knowledge, Perceptions, Resources, and Advice Given by Certified CrossFit Trainers

**DOI:** 10.3390/sports5020021

**Published:** 2017-03-24

**Authors:** Cassie Maxwell, Kyle Ruth, Carol Friesen

**Affiliations:** 1 Department of Nutrition and Health Science, College of Health, Ball State University, Muncie, IN 47306, USA ; cmaxwell@bsu.edu; 2 Training Think Tank, 11445 N Fulton Industrial Blvd Alpharetta, GA 30009, USA; kyle.ruth@trainingthinktank.com

**Keywords:** CrossFit trainers, sports nutrition knowledge, Paleo diet, Registered Dietitians (RD), Board Certified Specialist in Sports Dietetics (CSSD)

## Abstract

Background: CrossFit is a large, growing force in the fitness community. Currently, Level 1 and 2 CrossFit certification classes do not include nutrition education. The purpose of this study was to identify sports nutrition knowledge, perceptions, resources, and advice given by Certified CrossFit Trainers. Methods: An online questionnaire that measured these four constructs was placed on a private Facebook community, open only to certified CrossFit trainers, for 10 days. Results: Complete surveys were obtained from 289 CrossFit trainers. The mean Sport Nutrition Knowledge (SNK) score was 11.1 ± 2.1, equivalent to 65.3% ± 12.4% correct. The trainers perceived nutrition to be extremely important to athletic performance (9.4 ± 0.9 on a 10 point scale). Overall, the trainers graded their SNK higher than that of their CrossFit peers. The internet and CrossFit peers were the most frequently reported sources for nutrition information; Registered Dietitians were the least reported source. The Paleo and Zone diets were the most common dietary regimens recommended by CrossFit trainers. Results indicated a positive correlation between a CrossFit trainer’s self-reported hours of nutrition education and their SNK score (r = 0.17; *p* < 0.01). Conclusion: Nutrition education modules for Level 1 and 2 CrossFit trainers, developed with input from Board Certified Specialists in Sports Dietetics, are recommended.

## 1. Introduction

CrossFit, a fitness regimen developed by Greg Glassman, combines cardiovascular exercises, gymnastics, body weight movements, and weightlifting, all performed at high intensities [1,2,3]. Unlike other regimens, CrossFit turns fitness into a competitive sport, a relatively new concept in fitness [2]. Since 2013, the number of CrossFit affiliates has more than doubled, increasing from 5000 to more than 13,000, with well over 300,000 CrossFit participants worldwide (https://www.crossfit.com/). Due to its increasing popularity, CrossFit is recognized as a large, growing force in the fitness community. 

To meet demands, CrossFit provides certification classes for four levels of trainers [3]. The introductory certification course, CrossFit Level 1 Trainer (CF-L1), is a two-day introduction to methodology, concepts, and movements that commences with a proctored, closed book test containing 55 multiple-choice questions that requires a 70% score for passing. To achieve Level 2 Trainer (CF-L2) recognition, trainers must have a CF-L1 certificate, have at least six months of CrossFit training experience, and successfully complete the Level 2 Certificate Course, an intermediate-level seminar that builds on the concepts and movements introduced at the Level 1 course. Completion of the third course results in Certified CrossFit Level 3 (CF-L3) credentials. In addition to CF-L1 and CF-L2 requirements, CF-L3 candidates must pass the Certified CrossFit Trainer (CCFT) examination, which is accredited by the American National Standards Institute (ANSI). Trainers pursuing CF-L3 designation receive nutrition education with an emphasis on the Paleo diet. To maintain a CF-L3 certification, trainers must renew their current CPR/AED certification, acquire 50 CEUs, and document 900 CrossFit training hours following every 3-year term [3]. The fourth course, Certified CrossFit Level 4 Coach (CF-L4), is the culmination of CrossFit training. To be eligible, CrossFit trainers must demonstrate effective coaching skills by passing a performance evaluation. CrossFit trainers are evaluated against six criteria: teaching, seeing, correcting, group management, presence and attitude, and demonstration. Completion of CF-L4 results in Certified CrossFit Coach (CCFC) designation [3]. 

“Sports nutrition,” a subsect of nutrition, is specifically designed to cater to the nutritional needs of athletes [4]. Athletes, in their quest for ways to enhance their performance, often turn to nutrition to gain a competitive edge. The timing of intake and the quality of foods consumed before, during, and after exercise can impact performance, making nutrition an integral part of an athlete’s success [5]. With a greater understanding of the role of nutrition in sport, and a need for specialists in the field who can provide nutrition education specific for athletic performance, a certification process to prepare Board Certified Specialist in Sports Dietetics (CSSD) was established [6]. 

Although there is an increased awareness of the effect of nutrition on athletic performance, research indicates a large gap in nutrition knowledge among athletes at all levels, from elite athletes [7,8], to college athletes [9,10,11,12], to coaches and trainers [13,14,15]. In general, the literature indicate sport nutrition knowledge scores, obtained using a variety of instruments, range from 36%–73% correct. The inadequacy of nutrition knowledge covers a broad range of topics such as weight control, dietary supplements, and overall general nutrition information [7,8,9,10,11,12,13,14,15]. A recent systematic review of athletes’ and coaches’ nutrition knowledge concluded that a new, universal, up-to-date, validated measure of general and sports nutrition knowledge is needed to adequately measure sports nutrition knowledge [16].

Level 1 and 2 CrossFit trainers are not required to have any nutrition education. Level 3 and Level 4 CrossFit trainers obtain nutrition information during the certification process, but, despite a dearth of evidence in the literature indicating its effectiveness in enhancing performance, the training emphasizes use of the Paleolithic diet, a diet described by dietetic professionals as “debatable” at best [17]. Due to the increasing popularity of CrossFit training, along with the vital role of nutrition in athletic performance, assessing the sports nutrition knowledge of CrossFit trainers is warranted. The results of this study can be used to lay the foundation for the development of a working relationship between the CrossFit community and Board Certified Specialists in Sports Dietetics (CSSD) or Registered Dietitians (RD).

## 2. Methods 

### 2.1. Institutional Review Board

This study was deemed exempt by the Ball State University Institutional Review Board prior to implementation. The researchers conducting this study completed the Collaborative Institutional Training Initiative training.

### 2.2. Subjects

The population for this study included all 8875 CrossFit trainers 18 years of age or older who were members of a private Facebook Group *CrossFit Affiliate Owners* open only to certified CrossFit trainers. The Creative Research System’s Sample Size Calculator estimated 260 completed responses were needed to obtain a 95% confidence level and a confidence interval (margin of error) of 6 (http://www.surveysystem.com/sscalc.htm). Kyle Ruth, head coach of Training Think Tank, Alpharetta, GA, placed the Qualtrics© survey link on the private CrossFit Facebook site. Participants provided electronic consent prior to taking the survey. The request to complete the survey was posted twice over a period of ten days in March of 2016.

### 2.3. Instruments

An online survey instrument (available from the corresponding author) was designed by the authors to include four primary constructs: (1) nutrition perceptions; (2) basic sports nutrition knowledge (17 SNK questions); (3) nutrition resources used; and (4) types of dietary advice given by CrossFit trainers to their clients. The survey was distributed through Qualtrics©, an online survey platform (www.qualtrics.com). Participants rated their perception of nutrition’s role in overall well-being on a scale of 1 to 10, with 1 representing not important and 10 representing extremely important. Participants were asked to ‘grade’ both their and other CrossFit trainers’ sports nutrition knowledge on a 13 point scale ranging from A+ (scored as 13) to F (scored as 1). Basic sports nutrition knowledge was assessed with 17 questions that addressed four fundamental sport-nutrition constructs (hydration, energy needs/recovery, macronutrients, and micronutrients). These questions were previously validated in a study that examined sports nutrition knowledge of Division I athletes, coaches, and athletic trainers at a Midwestern University (manuscript in process). Because CrossFit promotes the Paleo diet, knowledge of the Paleo diet was also assessed. The frequency with which trainers used various resources to obtain nutrition information (i.e., other CrossFit trainers, Registered Dietitians (RD), the Internet, peer-reviewed research, and coaches/athletic trainers) was assessed using a 3-point Likert scale (Always, Sometimes, Never). Lastly, trainers were asked to indicate which of six common dietary regimens they recommend to their clients.

### 2.4. Statistical Analysis

Data was analyzed using SPSS v.23.0 for Windows (SPSS, 2016, IBM Corp., Armonk, NY, USA). Descriptive statistics and frequency counts (number and percent) were run on all variables. A corresponding variable was created for each of the 17 nutrition knowledge questions that indicated if the answer to the question was ‘right’ or ‘wrong’. The number of ‘right’ answers was summed to create an overall sport nutrition knowledge score (SNK), as well as sub-scores for each of the four nutrition constructs (i.e., hydration, energy needs/recovery, macronutrients, and micronutrients). The percent of correct nutrition knowledge questions was calculated. A Shapiro-Wilk test was run to test the normality of the data. Mean and median SNK scores were calculated for each CrossFit certification level. A Kruskal Wallis test was run to determine if there was a difference in SNK by group. Pearson’s correlation coefficient was used to examine the relationship between the SNK score by hours of nutrition education, perceived nutrition ‘grade’ of CrossFit trainers, and the individual’s perceived ‘grade’ of their own nutrition knowledge.

## 3. Results

### 3.1. Subjects

Between March 21 and March 30, 2016, a total 553 individuals clicked the link to the CrossFit Sport Nutrition Knowledge (SNK) survey that had been placed on a closed Facebook community available only to certified CrossFit trainers. Of these, 289 subjects completed the survey and submitted their answers. Survey analytics indicate the individuals who completed the survey spent an average of 10.3 ± 10.8 min; in contrast, the 274 individuals who clicked on the link but did not complete the survey only spent an average of 1.6 ± 2.3 min on the website. Of these, 43% spent less than 30 s on the survey site. 

By gender, 59.5% of the 289 responders (n = 172) were male and 40.5% (n = 117) were female. Respondents ranged in age from 19 to 63 years (mean age: 34.6 ± 8.4). Nine percent (n = 26) had completed high school or less, 17% completed an associate’s degree, more than half (53.5%) had completed a bachelor’s degree, 17.4% completed a master’s degree, and 3.1% of the respondents had completed a doctoral degree. Ninety-eight percent (n = 282) of the respondents provided their CrossFit Certification level. The majority of responders were CrossFit Level 1 certified (78.4%). One of every five (20.6%) responders was Level 2 certified. Only 1.1% of the respondents had Level 3 certification. No Level 4 CrossFit certified trainers participated in the present study (Table 1).

### 3.2. Perceptions of CrossFit Trainers

The CrossFit trainers indicated how important they perceived “nutrition” was to athletic performance using a Likert scale ranging from one to ten, where 1 was “not important” and 10 was “extremely important.” The mean score of 9.4 ± 0.9 indicates the CrossFit trainers perceived nutrition as being extremely important to athletic performance. The trainers were asked what ‘grade’ they would give the sports nutrition knowledge of most CrossFit trainers. Options ranged from A+ (scored as 13) to F (scored as 1). While the trainers gave their peers an average grade of C+ (7.6 ± 2.1) (n = 285), when asked to grade their own sports nutrition knowledge, the trainers gave themselves an average grade of B+ (9.6 ± 1.7) (n = 269), significantly higher than the grade they gave their peers (t = 14.8; *p* < 0.001).

### 3.3. Nutrition Knowledge of CrossFit Trainers

**Figure 1:**
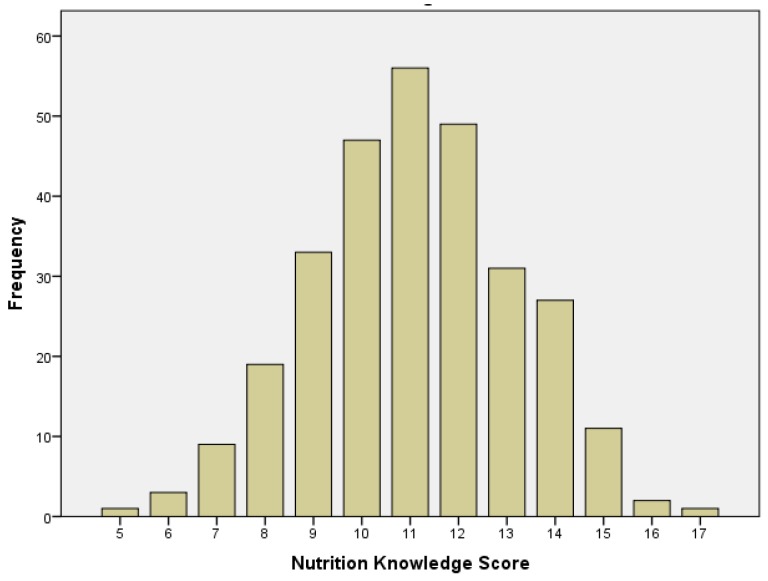
Frequency of Nutrition Knowledge Score (Range 5–17) (N = 289).

In this study, the construct “sports nutrition knowledge” was measured using 17 questions adapted from Shapiro et al. [18]. The number of correct answers were summed to calculate the overall sports nutrition knowledge (SNK) score. Scores ranged from a low of 5 (29% correct) to a high of 17 (100% correct) (Figure 1). The median SNK score among the 289 respondents was 11; the mean SNK was 11.1 ± 2.1, equivalent to answering 65.3% ± 12.4% of the questions correctly (Table 2).

The 17 SNK questions were categorized into four basic sports nutrition constructs: (1) hydration; (2) energy needs/recovery; (3) macronutrients; and (4) micronutrients. By construct, results indicate the respondents were the most knowledgeable about energy needs/recovery (75% correct) and micronutrients (70% correct), but less knowledgeable about hydration (55% correct) and macronutrients (50% correct) (Table 2). The Kruskal Wallis test indicated no difference in the SNK by CrossFit Certification Level (*X*^2^ = 3.347; *p* = 0.188).

### 3.4. Resources Used for Nutrition Information by CrossFit Trainers

The CrossFit trainers used a 3-point Likert scale (Always = 1, Sometimes = 2, and Never = 3) to indicate what resources they used to obtain nutrition information from a list of five potential options. Overall, the internet was cited as the most frequent resource used by the CrossFit trainers (1.92 ± 0.54), followed by peer-reviewed research articles (1.93 ± 0.59), and other CrossFit colleagues (1.98 ± 0.49). In contrast, Registered Dietitians were cited by the CrossFit trainers as the least used resource to obtain nutrition information (2.13 ± 0.62), with more than one-quarter (26%) of the respondents indicating they had never used a Registered Dietitian as a resource of nutrition information (Table 3). 

### 3.5. Relationship between Sports Nutrition Knowledge and Nutrition Education

The CrossFit trainers estimated they had received, on average, 37.2 ± 27.6 h of nutrition education, with the number of hours ranging from 0 (n = 5) to more than 100 (n = 19) hours. There was a positive correlation between the number of self-reported hours of nutrition education and the overall sports SNK (r = 0.19; *p* < 0.01), although the effect size was weak to moderate. 

### 3.6. Dietary Recommendations Given by CrossFit Trainers

Respondents were asked to indicate what dietary regimens they typically recommend to their clients (Table 4). The two most common dietary regimens recommended were the Paleo (40%) and Zone (44%) diets. A wide variety of ‘other’ dietary recommendations made by the trainers included drinking more water (n = 8), eliminating sugar (n = 6), eating clean (n = 5) and “if you can’t spell it, don’t eat it!”(n = 1).

## 4. Discussion

### 4.1. Perception of the Importance of Nutrition among CrossFit Trainers

Research has documented the critical importance of adequate nutrition to athletic performance [5]. Results of the present study indicate certified CrossFit trainers also perceive nutrition to be extremely important to athletic performance. Although believed to be important, the trainers appeared to have qualms about the nutrition knowledge of their peers, with the trainers giving their peers a lower nutrition knowledge grade (C+) than they gave to themselves (B+). The discord between the trainers’ belief in the importance of nutrition and their perception of the nutrition knowledge of their peers and themselves, suggests the trainers would benefit if nutrition education was included as part of the Level 1 and Level 2 CrossFit certification process. 

### 4.2. Nutrition Knowledge of CrossFit Trainers

The average sports SNK score in this study, equivalent to answering 65% of the questions correctly, is equivalent to receiving a ‘D’ letter grade when using the standard American grading scale. Interestingly, this score is significantly lower than the grade the trainers assigned to their peers (C+) or to themselves (B+), suggesting the trainers overestimated their own sports nutrition knowledge as well as that of their peers. These results provide more evidence to indicate the CrossFit trainers would benefit from targeted nutrition education.

Although no study was identified in the literature that examined the sport nutrition knowledge of CrossFit trainers, the nutrition knowledge of various types and levels of athletes and coaches has been examined by many researchers. Among elite athletes, Alaunyte et al. reported a relatively high mean correct nutrition knowledge score of 72.8% ± 6.11 (out of 100) among elite rugby players [7] while Devlin et al., reported a mean nutrition score of 60.5% (no SD provided) among elite Australian football players [8]. Among collegiate athletes, Torres-McGehee et al. reported a mean SNK of 54.9% ± 13.5% [12], Shapiro reported a mean SNK of 67.9% ± 19.4% [18], and Hornstrom et al. reported a mean SNK for MAC softball athletes of 57.1% ± 5.8% [10]. Several researchers have examined the SNK of collegiate coaches, athletic trainers, and strength and conditioning specialists. Barbaros-Tudor et al. reported a mean SNK of 68.9% (SD not reported) in a survey of collegiate coaches [13]. Similarly, Botsis and Holden reported a mean SNK of 55% (SD not reported) among collegiate coaches [14]. Torres-McGehee et al. reported a mean SNK of 65.9% ± 14.3%, 77.8% ± 10.3%, and 81.6% ± 10.3% among college coaches, athletic trainers, and strength and conditioning specialist, respectively [12]. In sum, the SNK of the CrossFit trainers in the present study (65.3% ± 12.4%) are similar to the SNK scores reported by previous researchers among collegiate athletes and coaches, but and lower than those reported among athletic trainers and strength and conditioning coaches. It is imperative to remember that a variety of instruments were used in each of these studies to assess SNK. Trakman et al. recently concluded that previous reports of nutritional knowledge needs to be interpreted with caution until a new, universal, up-to-date, validated measure of general and sports nutrition knowledge instrument is developed [16]. 

By construct, ‘energy needs/recovery’ was the most well-understood sports nutrition-related topic among the CrossFit trainers with a mean score of 75% correct. Shapiro reported student-athletes answered 60% of recovery nutrition and timing questions correctly [18]. Due to the demanding nature of CrossFit training, it is crucial that CrossFit clients properly refuel their body in order to build their depleted glycogen stores, reduce muscle breakdown, and promote muscle recovery. CrossFit trainers answered approximately half of hydration questions correctly. Torres et al. reported a similar hydration score (54.7%) among collegiate athletes; however, the athletic trainers scored higher significantly higher (79.4%; *p* < 0.05) [12]. Shapiro reported student-athletes answered 48% of hydration questions correctly [18]. CrossFit trainers answered micronutrient and macronutrient questions with a proficiency of 70% and 50%, respectively, consistent with other research. Torres grouped micro- and macronutrients into one construct, with athletes scoring 51.8% correct while athletic trainers scored 70.7% correct [12]. Based on the present study, the results indicate Level 1 and 2 CrossFit trainers would benefit from nutrition education, particularly about macronutrient distribution (i.e., carbohydrate, protein and lipids) to meet the bodies need for muscle growth and energy. 

There was no significant difference between CrossFit Certification Levels 1–3 and any of the four constructs. A requirement of CrossFit Level 3 (CF-L3) certification is mandatory nutrition education. As such, it was expected that their nutrition knowledge, both overall and by construct, would be significantly higher than their CF-L1 and CF-L2 peers. However, because only 1.1% (n = 3) of participants in the present study were CF-L3 trainers (n = 282), these findings cannot be considered representative of the entire CrossFit trainer population. Repeating this study with an emphasis on recruiting CF-L3 and CF-L4 trainers is warranted.

### 4.3. Dietary Recommendations Given by CrossFit Trainers

CrossFit trainers indicated they recommend the Paleo and Zone diets the most frequently. This was not surprising since the CrossFit website (https://www.crossfit.com/) emphasizes the use of, both, the Paleo and Zone diets. The Paleolithic (Paleo) diet differs radically from dietary patterns currently recommended in guidelines, particularly in terms of its recommendation to exclude grains, dairy, and nutritional products of industry [19]. While a recent systematic review of the Paleo diet concluded that the Paleolithic diet resulted in greater short-term improvements in metabolic syndrome components than did guideline-based control diets [19], no controlled studies have been identified in the literature that indicate the Paleo diet enhances athletic performance. Research that examines the sufficiency of the Paleo diet in meeting the nutritional requirements of CrossFit participants is needed.

### 4.4. Resources Used for Nutrition Information by CrossFit Trainers

Eighty-nine percent of the participants in this study indicated that they ‘always’ or ‘sometimes’ used the internet to obtain nutrition information. In contrast, Registered Dietitians were the least used resource, with more than one-quarter (26%) of the CrossFit trainers indicating they had never used a Registered Dietitian as a resource for nutrition information. It is the position of the Academy of Nutrition and Dietetics, Dietitians of Canada, and the American College of Sports Medicine that athletes be referred to a Registered Dietitian for a personalized nutrition plan [5]. CrossFit participants should be encouraged to collaborate with Registered Dietitians in an effort to ensure that they meet their nutritional needs. In addition, CSSDs and RDs should collaborate with leaders in the CrossFit community to develop nutrition education modules for inclusion in the Level 1 and Level 2 CrossFit certification classes.

### 4.5. Limitations and Future Suggestions

There are several limitations of this study. First, although the survey was placed on a private Facebook site established for Certified CrossFit Trainers, one cannot assume that the anonymous responders were, indeed, certified trainers. In addition, although previously validated and tested for reliability, the brief 17-question survey may not provide adequate coverage of sport-nutrition concepts to be able to discern a difference in the sport nutrition knowledge of the CrossFit trainers who completed the survey. The low number of Level 3, and the dearth of Level 4, CrossFit trainers who completed the survey prohibits identifying the SNK score among the more highly trained CrossFit trainers. Lastly, without additional research, these findings cannot be considered representative of the entire CrossFit trainer population. 

Nonetheless, the results of this study provide support for the development and inclusion of nutrition education modules in the Level 1 and Level 2 CrossFit classes. Board Certified Specialists in Sports Dietetics (CSSD) should be encouraged to work in collaboration with the leaders of CrossFit.org to develop appropriate nutrition education materials. In addition to the training modules, supplemental materials containing reliable references, nutrition guidelines, and unique requirements of athletes could be created to distribute to CrossFit trainers for their professional and personal use. Future studies should examine the differences in nutrition knowledge across CrossFit certification levels. To do so, a larger sample of CF-L3 and CF-L4 trainers should be obtained. 

## 5. Conclusions

CrossFit training is a demanding fitness regimen that incorporates a variety of functional movements performed at high intensity (https://www.crossfit.com). For optimal performance, it is critical that CrossFit athletes obtain nutrition education and receive appropriate nutrition counseling. At present, CrossFit trainers do not receive nutrition education until the CrossFit Level 3 (CF-L3) certification. The results of this study indicated that, while CrossFit trainers perceive nutrition to be important to athletic performance, their nutrition knowledge was not optimal. The positive correlation observed between the number of self-reported hours of nutrition education and the overall SNK, indicates nutrition education would be beneficial for CrossFit trainers. Despite being experts in nutrition, Registered Dietitians (RD) or Board Certified Specialists in Sports Dietetics (CSSD) were not perceived as a primary source for sports nutrition information in the present study. Collaborating with an RD or CSSD may be beneficial, particularly for CF-L1 and CF-L2 certified trainers, to optimize one’s nutrition knowledge and, ultimately, an individuals’ athletic performance.

## Figures and Tables

**Table 1:** Respondents by CrossFit Certification Level (n = 282).

CrossFit Certification Level	Number of Respondents	Percent of Respondents
Level 1	221	78.4
Level 2	58	20.6
Level 3	3	1.1
Level 4	0	0.0

**Table 2:** Sports Nutrition Knowledge Score, Overall and by Construct (n = 289).

SNK Overall and by Construct	Number of Questions	Mean ± SD	Percent Correct
Total Overall SNK	17	11.1 ± 2.1	65%
SNK Construct Sub-Scores			
Energy Needs/Recovery	4	3.0 ± 0.8	75%
Micronutrients	7	4.9 ± 1.1	70%
Hydration	2	1.1 ± 0.7	55%
Macronutrients	4	2.0 ± 1.2	50%

**Table 3:** Frequency with which Resources were used by CrossFit Trainers to Obtain Nutrition Information.

Resource	N	Always (1)	Sometimes (2)	Never (3)	Mean ± SD
Internet	284	55 (19%)	198 (70%)	31 (11%)	1.92 ± 0.54
Peer-reviewed research	279	59 (21%)	180 (65%)	40 (14%)	1.93 ± 0.59
Other CrossFit colleagues	284	36 (13%)	217 (76%)	31 (11%)	1.98 ± 0.49
Former coaches/trainers	282	41 (15%)	198 (70%)	43 (15%)	2.01 ± 0.55
Registered Dietitians	284	39 (14%)	170 (60%)	75 (26%)	2.13 ± 0.62

**Table 4:** Dietary Recommendations Given by CrossFit Trainers.

Diet Regimen	Number of Respondents	Percent
Zone diet	128	44
Paleo diet	115	40
Other	114	39
Gluten free diet	43	15
No recommendations given	36	12
Mediterranean diet	19	7
Vegan diet	3	1
DASH diet	0	0
